# A Highly Sensitive Electrochemical Immunosensor Based on Core–Shell SiO_2_-PEI@Ag NPs Nanocomposites for MPA Detection

**DOI:** 10.3390/foods15152701

**Published:** 2026-07-31

**Authors:** Yadi Yuan, Jingming Zhou, Hongliang Liu, Haili Wang, Yanhua Qi, Enping Liu, Yongxin Li, Aiping Wang

**Affiliations:** 1 School of Life Sciences, Zhengzhou University, Zhengzhou 450001, China; 2 Longhu Laboratory of Advanced Immunology, Zhengzhou 450046, China; 3 Henan Provincial Key Laboratory of Immunobiology, Zhengzhou 450001, China

**Keywords:** SiO_2_-PEI@Ag NPs nanocomposites, electrochemical immunosensor, MPA

## Abstract

In recent years, food safety concerns associated with veterinary drug residues have aroused significant public attention, highlighting the critical need for continuous monitoring in both animal-derived foods and the environment to safeguard public health. Medroxyprogesterone acetate (MPA), a major anabolic steroid widely used in animal husbandry, poses potential human health risks according to recent studies. Although conventional analytical techniques based on chromatography and immunological assays are effective, they often suffer from complex operations, the need for skilled personnel, high cost, and time consumption. To overcome these limitations, electrochemical immunosensors modified with advanced nanomaterials offer a promising alternative. Herein, a highly sensitive electrochemical immunosensor was developed for trace MPA detection utilizing core–shell SiO_2_-PEI@Ag nanoparticles as the sensing matrix. In this nanocomposite, the structural synergy between the SiO_2_ core and outer Ag NPs shell not only provides a high-density platform for specific antibody immobilization, but also drastically expands the electroactive surface area and accelerates electron transfer to maximize signal amplification. Benefiting from these synergistic advantages, the constructed immunosensor exhibited a limit of detection (LOD) of 0.014 ng/mL and a wide linear range of 0.03–100 ng/mL. Furthermore, the recovery rates in pork and milk samples were 91.32–97.29% and 95.14–102.54%, respectively. In addition, negligible cross-reaction was observed toward structurally related steroid hormones related to MPA and common antibiotics. The immunosensor shows good stability, reproducibility, and accuracy, demonstrating great potential as a promising candidate to complement conventional tools for future MPA residue screening.

## 1. Introduction

Progesterone is a 21-carbon steroid hormone synthesized primarily in the adrenal cortex and the corpus luteum; it supports embryo implantation, maintains pregnancy, and functions as the primary luteal-phase supplement in assisted reproductive technologies [1,2]. Natural progesterone and numerous synthetic progestins are increasingly used in contraception and medicinal therapies in both humans and animals [3,4,5]. Among these synthetic progestins, Medroxyprogesterone acetate (MPA) is a 17-hydroxyprogesterone derivative that differs from progesterone by the presence of a methyl group at carbon 6 and an acetate group at carbon 17 [6,7]. These structural modifications substantially enhance its oral progestational potency and bioavailability. Due to these advantageous features, MPA has been widely employed worldwide for estrus induction and synchronization, as well as a growth-promoting agent to accelerate fattening in livestock production [8,9,10].

However, the widespread use of MPA has also led to illegal abuse and food safety concerns, highlighted by the notorious MPA contamination incident in the European Union in 2002. These concerns are deeply rooted in its severe toxicological risks, the International Agency for Research on Cancer classified MPA as a Group 2B carcinogen in 2007, and recent epidemiologic studies link MPA exposure to increased risks of meningioma, cardiovascular disorders, and potential neurological effects [11,12,13,14]. Furthermore, prolonged intake of progesterone analogs, even at low concentrations, can disrupt endocrine and immune homeostasis. Consequently, the use of MPA and certain progestins in livestock has been banned or strictly regulated with defined maximum residue limits. Therefore, establishing sensitive and reliable analytical methods is crucial for detecting MPA residues in animal-derived foods.

In recent decades, various analytical methods have been developed for MPA detection [15,16,17,18,19]. Specifically, the National Food Safety standard (GB 31660.4-2019 [20]) designates liquid chromatography-tandem mass spectrometry (LC-MS/MS) as the official method for detecting MPA residues in animal-derived foods, with a limit of detection (LOD) of 0.5 μg/kg. Chromatography is still commonly used for monitoring veterinary drug residues because of its high sensitivity and accuracy. However, it typically requires expensive instrumentation, cumbersome sample pre-processing steps involving hazardous organic solvent extraction to remove complex endogenous interfering substances, and the continuous consumption of costly mobile phases. Furthermore, professional training is strictly required for researchers to operate these sophisticated instrumentations. In contrast, enzyme-linked immunosorbent assays (ELISA) and immunochromatographic strip assays have the advantages of low detection costs, minimal simple procedure, and simple operational procedures relative to chromatographic methods, enabling the efficient processing of large sample batches for rapid screening. Nevertheless, these conventional immunoassays suffer from insufficient sensitivity for trace residue determination, with ELISAs additionally requiring prolonged incubation times and labor-intensive washing, and rapid strips lacking precise quantitative capability [21,22,23]. Therefore, advanced biosensor technologies featuring low cost, simple operation, and high sensitivity have attracted widespread attention, with various promising strategies being actively explored [24,25,26].

Among these novel platforms, electrochemical biosensors, characterized by the integration of biological components and advanced nanomaterials, have received much attention in the fields of biomarker and drug residue detection [27,28,29,30]. However, electrochemical biosensors for MPA detection in animal-derived foods have not yet been reported. In terms of nanomaterial selection for sensor fabrication, gold and silver nanoparticles (Au/Ag NPs) are generally preferred due to their excellent electrical conductivity and catalytic activity. Notably, Ag NPs exhibit enhanced performance over gold due to their lower reaction potential. Concurrently, Ag NPs are characteristically easier to prepare and more cost-effective than Au NPs, making them a highly advantageous choice [30,31,32,33]. Nevertheless, the intrinsic electrochemical performance of pristine Ag NPs cannot be fully harnessed due to their chemical instability and high propensity for aggregation [30,32]. To address this issue, the introduction of core–shell structured nanoparticles represents a highly effective strategy, where these composite materials fully capitalize on the complementary strengths of different material phases via a synergistic mechanism, thereby substantially expanding the electroactive surface area and subsequently boosting the overall analytical performance of the resulting sensors [34,35,36].

Typically, a diverse library of individual components, which include metals, two-dimensional structures, and porous nanomaterials, can be utilized for core–shell fabrication. Distinct from graphene and metal–organic frameworks (MOFs) that often suffer from structural restacking and non-uniform dimensions, respectively, silicon dioxide nanoparticles (SiO_2_ NPs) with high porosity have been widely utilized in sensing systems, owing to their ease of synthesis, uniform pore size, exceptional colloidal stability, ease of surface functionalization, and outstanding biocompatibility [37,38,39]. Upon surface modification, the silica cores can assemble with metal nanoparticles through covalent bonding or physical adsorption to form stable nanocomposites. Building on these advantages, an electrochemical immunosensor based on SiO_2_-PEI@Ag NPs and anti-MPA antibody was developed for the determination of MPA residues in animal-derived foods. The proposed sensor was successfully validated with MPA-spiked pork and milk samples, exhibiting satisfactory recoveries and serving as a promising analytical prototype that could be extended to practical food safety screening in the future.

## 2. Materials and Methods

### 2.1. Materials

Tetraethyl orthosilicate (TEOS) and sodium citrate dihydrate were bought from Aladdin Chemical Co., Ltd. (Shanghai, China). BSA and aqueous ammonia were acquired from Sigma-Aldrich (St. Louis, MO, USA). AgNO_3_ was bought from Sinopharm Chemical Reagent Co., Ltd. (Shanghai, China). Sodium bromide (NaBr) and ethylene imine polymer (PEI) were bought from Macklin Biochemical Co., Ltd. (Shanghai, China). Medroxyprogesterone acetate (MPA, Cat No. SYY27920) and ascorbic acid (AA) were purchased from Solarbio Science and Technology Co., Ltd. (Beijing, China). Milli-Q ultrapure water (18.2 MΩ·cm^−1^) was employed for all solution preparations. The 0.01 M phosphate-buffered saline (PBS) buffer was prepared by dissolving 2.9 g of Na_2_HPO_4_·12H_2_O, 0.2 g of KC1, 8 g of NaCl, and 0.2 g of KH_2_PO_4_ to a final volume of 1 L. Similarly, the 0.1 M PBS buffer was prepared by dissolving 21.95 g of Na_2_HPO_4_·12H_2_O, 7.45 g of KC1, and 5.267 g of KH_2_PO_4_ to a final volume of 1 L.

Anti-MPA monoclonal antibody (mAb, clone 6E2) was developed in-house by our laboratory via standard hybridoma technology and purified from mouse ascites using the caprylic acid-ammonium sulfate method. Characterization demonstrated that mAb 6E2 possessed a high titer in ascites of 1:2.048 × 10^6^, an affinity constant (Kaff) of 2.75 × 10^8^ L/mol, and an IC_50_ value of 0.26 ng/mL toward MPA, with low cross-reactivity (<8%) against structural analogues including Flurogestone acetate (Cat No. SF9550, Solarbio), Testosterone (Cat No. SH9750, Solarbio), and 17-Methyltestosterone (Cat No. SYA17600, Solarbio).

### 2.2. Apparatus

The nanomaterials were characterized using a NanoDrop 2000c spectrophotometer (Thermo Fisher Scientific, Waltham, MA, USA), a Thermo Scientific Talos L120C TEM, an Auriga-bu focused ion beam scanning electron microscope equipped with EDS (Zeiss, Oberkochen, Germany), and an IRTracer-100 FT-IR spectrophotometer (Shimadzu, Kyoto, Japan). The surface charge of the nanoparticles was determined by zeta potential measurements using a Malvern Zetasizer Nano ZSP. All electrochemical measurements were carried out on a CHI660E workstation (Shanghai CH Instruments, Shanghai, China) using a conventional three-electrode system consisting of a modified gold electrode, a saturated calomel electrode, and a platinum wire serving as the working, reference, and counter electrodes, respectively.

### 2.3. Synthesis of Silver Nanoparticles

The monodisperse Ag NPs were synthesized utilizing a chemical reduction approach, as previously reported with minor modifications [40]. Briefly, 50 μL of AA (0.1 M) was added to 47.5 mL of boiling water and boiled for approximately 1–2 min. Concurrently, a precursor solution was prepared by homogenizing AgNO_3_ (1 mL, 2.5 mg/mL), sodium citrate (1 mL, 10 mg/mL), and NaBr (0.5 mL, 0.8 mg/mL) at ambient temperature for 5 min and then introduced into the boiling AA solution. The reaction mixture was maintained at boiling temperature for 1 h under continuous magnetic stirring at 600 rpm, accompanied by a rapid color change from colorless to yellow. Afterward, the mixture was cooled naturally to ambient temperature in a dark environment. The resulting Ag NPs were collected by centrifugation (10,000 rpm, 30 min), washed three times with ultrapure water, and then re-dispersed in 5 mL of ultrapure water. The final suspension was stored at 4 °C for further use.

### 2.4. Preparation of SiO_2_-PEI@Ag NPs Nanocomposites

**Figure 1:**
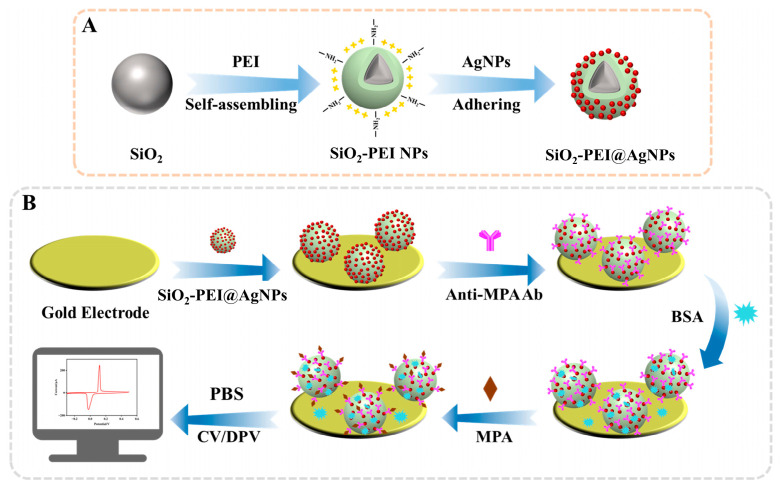
(**A**) Synthesis principle of SiO_2_-PEI@Ag NPs. (**B**) The schematic illustration for the fabrication of the MPA electrochemical immunosensor.

As illustrated in Figure 1A, the SiO_2_-PEI@Ag NPs nanocomposites were prepared based on previously reported methods with slight modifications [37,41,]. SiO_2_ nanoparticles were prepared by adding 4 mL of TEOS to a mixture of ethanol (100 mL), aqueous ammonia (4 mL), and ultrapure water (6 mL), followed by stirring at ambient temperature for 4 h (600 rpm). The resulting SiO_2_ NPs were collected by centrifugation at 6000 rpm for 5 min, washed five times with ultrapure water, and ultrasonically dispersed in 20 mL of ethanol for 30 min. Subsequently, the SiO_2_ NPs were functionalized with PEI by adding 2 mL of the ethanol suspension to an aqueous PEI solution (10 mg/mL), followed by sonication for 60 min in an ice-water bath to prevent overheating. After washing three times with ultrapure water, the resulting SiO_2_-PEI NPs were dispersed in 2 mL of ultrapure water and sonicated for 30 min. Finally, 0.06 mL of the resulting suspension was mixed with previously prepared Ag NPs (1.7 mL) to obtain SiO_2_-PEI@Ag nanocomposites, which were stored at 4 °C until further use.

### 2.5. Construction of an Electrochemical Immunosensor

The working electrodes were sequentially polished with 0.3 and 0.05 μm Al_2_O_3_ powders to obtain a mirror-like surface prior to modification. After each polishing stage, the electrodes were thoroughly rinsed and sonicated in absolute ethanol and ultrapure water for 1 min each. The stepwise assembly of the immunosensor was carried out as schematized in Figure 1B. The working electrode was first modified by drop-casting 10 μL of SiO_2_-PEI@Ag NPs suspension onto its surface and allowing it to air-dry at room temperature. The modified electrode was then incubated with 20 μL of anti-MPA antibody solution (diluted 1:1000 in 0.01 M PBS) at 37 °C for 45 min. After rinsing with 0.1 M PBS, the electrode was further incubated with 20 μL of BSA solution (0.5 mg/mL) at 37 °C for 60 min to block nonspecific binding sites. Finally, the prepared immunosensor was either used for MPA detection or stored at 4 °C until further use.

### 2.6. Electrochemical Measurements

The electrochemical behavior of the immunosensor was investigated using cyclic voltammetry (CV), electrochemical impedance spectroscopy (EIS), and differential pulse voltammetry (DPV). CV measurements were carried out in PBS (0.1 M, pH 7.4) over a potential range of −0.3 to 0.5 V at a scan rate of 100 mV·s^−1^. EIS analysis was conducted in PBS containing 5 mM [Fe(CN)_6_]^3−/4−^ over frequencies ranging from 0.1 Hz to 100 kHz, init E 0.199 V, amplitude 0.005 V, and quiet time 2 s. For the analytical evaluation of the immunosensor, DPV was performed in 0.1 M PBS with the potential varied between −0.08 and 0.26 V, incr E 0.004 V, amplitude 0.05 V, pulse width 0.05 s, sample width 0.0167 s, pulse period 0.5 s, and quiet time 2 s. To establish the calibration curve, the prepared immunosensors were incubated with 20 μL of MPA solutions at various concentrations for 60 min at 37 °C. The corresponding peak currents obtained from DPV measurements were used to construct the linear regression plot. Finally, pork and milk samples purchased from a local supermarket were used for spiked recovery assays, and DPV measurements were performed to assess the practical performance of the immunosensor in real samples. These samples were pretreated according to previously reported protocols [27].

## 3. Results and Discussion

### 3.1. Morphology and Physicochemical Properties of SiO_2_-PEI@Ag NPs

**Figure 2:**
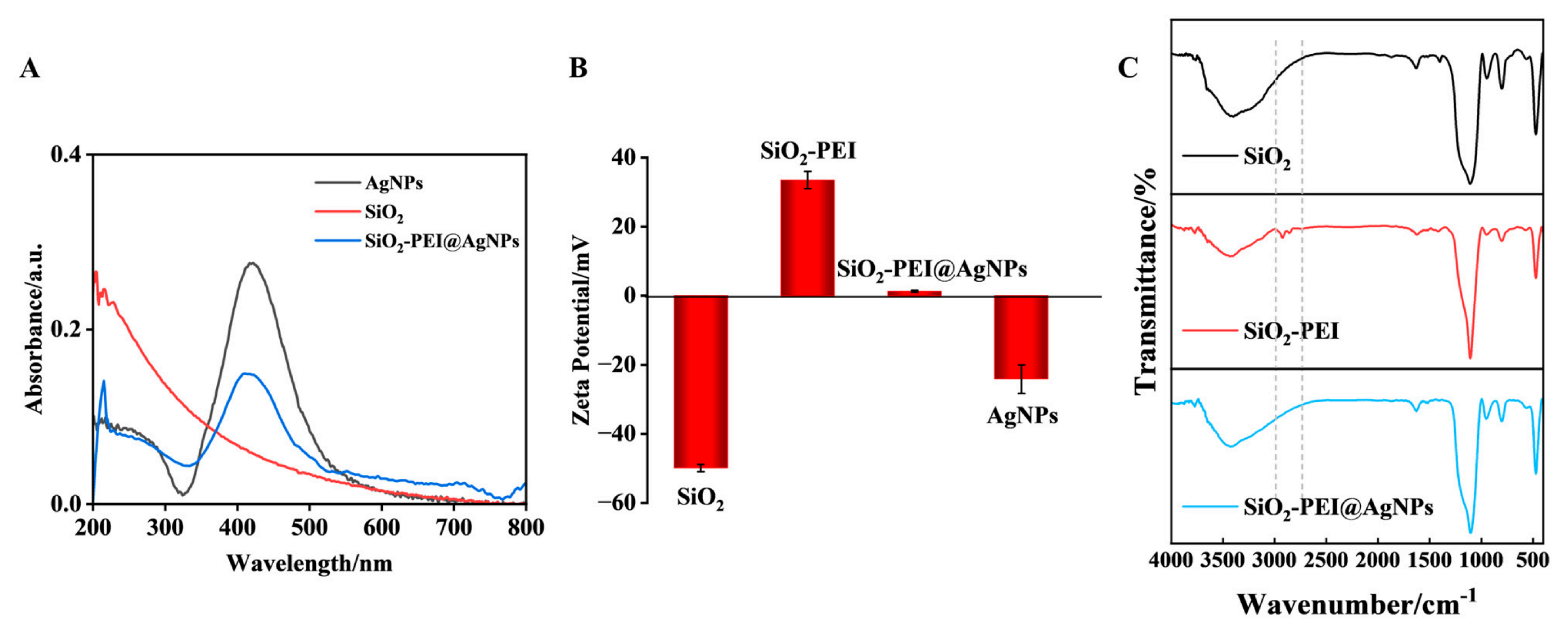
(**A**) UV-Vis spectra of Ag NPs, SiO_2_ and SiO_2_-PEI@Ag NPs. (**B**) Zeta potentials of SiO_2_, SiO_2_-PEI, SiO_2_-PEI@Ag NPs and Ag NPs. (**C**) FT-IR spectra of SiO_2_, SiO_2_-PEI and SiO_2_-PEI@Ag NPs. The vertical dashed lines are used as guides to indicate key characteristic peaks for comparison.

The SiO_2_-PEI@Ag NPs used in this investigation were successfully synthesized via a three-step PEI-mediated layer-by-layer assembly strategy (Figure 1A). The formation of the nanocomposites during stepwise surface modification was confirmed by UV-Vis, FT-IR and Zeta potential measurements. As shown in Figure 2A, SiO_2_ shows no absorption peak in the UV-Vis spectrum, whereas SiO_2_-PEI@Ag NPs and Ag NPs exhibit a distinct absorption peak at about 418 nm, which is attributed to the Mie plasmon resonance excitation from the silver nanoparticles [42]. Figure 2B shows that the Zeta potentials of SiO_2_, SiO_2_-PEI, SiO_2_-PEI@Ag NPs and Ag NPs in ultrapure water were −49.9, +33.5, +1.3 and −24.2 mV, respectively. The surface charge of SiO_2_ changed from negative to positive after PEI modification, indicating the successful assembly of the PEI layer on the silica surface. Subsequently, the decrease in zeta potential after Ag NPs loading suggested the attachment of Ag NPs onto the SiO_2_-PEI surface. These results demonstrate that the formation of SiO_2_-PEI@Ag NPs was driven by electrostatic adsorption and coordination interaction between Ag NPs and the PEI layer. In addition, Figure 2C presents the FT-IR spectra of SiO_2_, SiO_2_-PEI and SiO_2_-PEI@Ag NPs. Two new absorption bands at 2854 cm^−1^ and 2922 cm^−1^ appear in SiO_2_-PEI, which are assigned to the symmetric and asymmetric C-H stretching vibrations of PEI [43,44].

**Figure 3:**
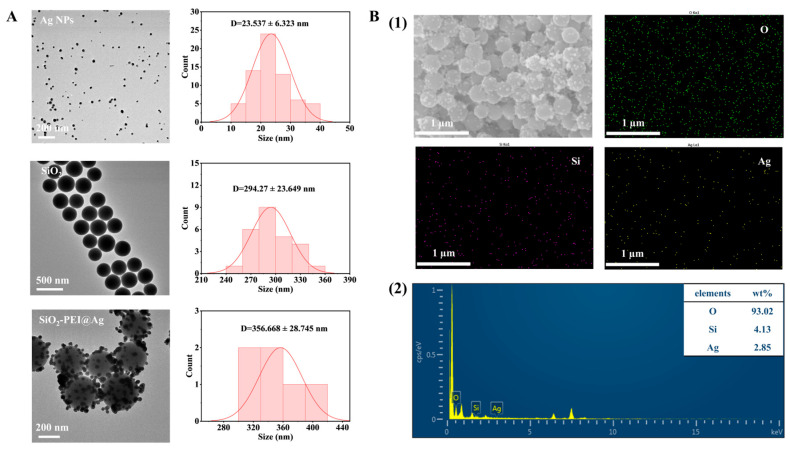
(**A**) TEM images and corresponding size distribution histograms of Ag NPs, SiO_2_, and SiO_2_-PEI@Ag NPs. (**B**) Characterization of SiO_2_-PEI @Ag NPs modified on the gold electrode. (1) SEM images, (2) EDS spectrum with element weight percentages.

TEM and SEM images revealed a nearly spherical morphology of the SiO_2_ core, with Ag NPs densely distributed on its surface (Figure 3). Subsequently, statistical analysis was performed using Image J software (version 1.54p, National Institutes of Health, Bethesda, MD, USA) to measure the particle size distributions. Figure 3A shows that the average diameters of the Ag NPs, SiO_2_ cores, and the resulting SiO_2_-PEI@Ag NPs nanocomposites were 23.537 ± 6.323 nm, 294.27 ± 23.649 nm, and 356.668 ± 28.745 nm, respectively. This integrated size profile well matched the theoretical expectation for a core–shell structure consisting of a central SiO_2_ core enclosed by an outer Ag NPs shell. Furthermore, the EDS spectrum and corresponding elemental mapping results confirmed that the nanocomposites mainly consisted of O, Si, and Ag elements, with measured weight percentages (wt%) of 93.02%, 4.13%, and 2.85%, respectively (Figure 3(B2)). These characterization results collectively confirmed the successful synthesis of SiO_2_-PEI@Ag NPs, enabling their practical deployment in subsequent immunosensor development (Figure 1B).

### 3.2. Electrochemical Characterization of the Immunosensor

**Figure 4:**
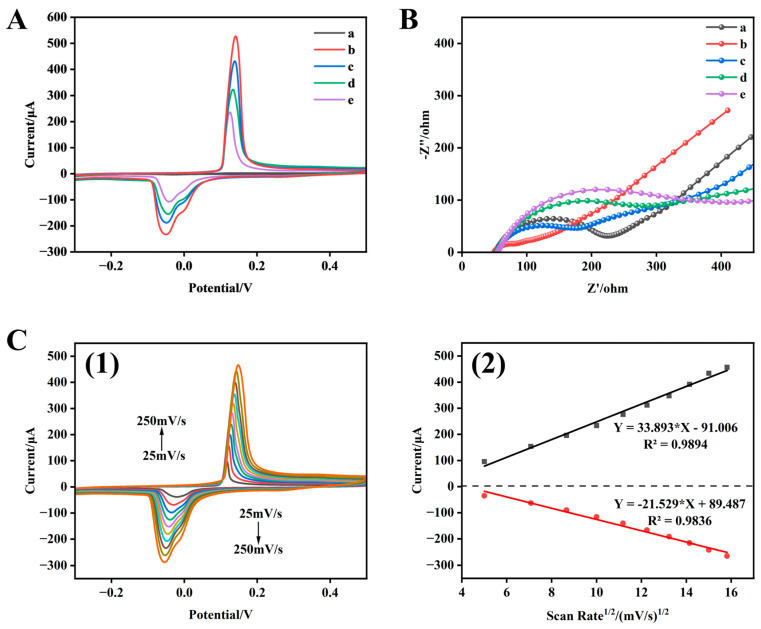
(**A**) CV and (**B**) EIS responses of the bare gold electrode (a), SiO_2_-PEI@Ag NPs (b), anti-MPA mAb/ SiO_2_-PEI@Ag NPs (c), BSA/anti-MPA mAb/ SiO_2_-PEI@Ag NPs (d), and MPA/BSA/anti-MPA mAb/ SiO_2_-PEI@Ag NPs (e). (**C**) CV responses of the immunosensor at different scan rates (25, 50, 75, 100, 125, 150, 175, 200, 225, 250 mV·s^−1^). (1) CV curves, where arrows indicate the trend of peak currents with increasing scan rates from 25 to 250 mV·s^−1^, (2) linear plots of redox peak currents versus scan rates.

**Figure 5:**
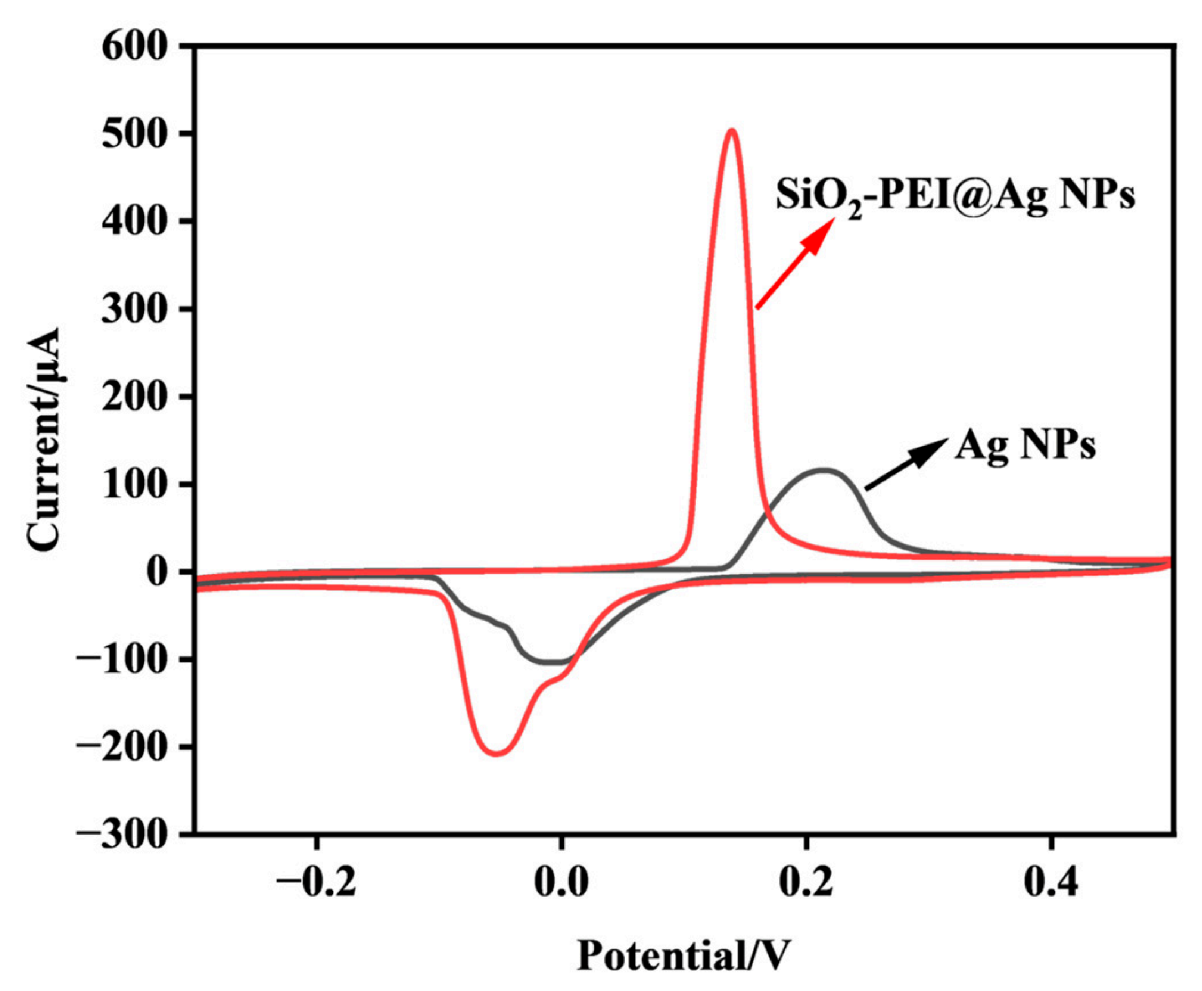
CV comparison of electrodes modified with pristine Ag NPs and SiO2-PEI@Ag NPs.

The assembly process of the immunosensor was characterized using CV to monitor the solid-state silver oxidation and reduction signals. As shown in Figure 4A, the bare gold electrode (curve (a)) exhibited almost no current signal when immersed in 0.1 M PBS (pH 7.4) due to the absence of electroactive redox couples. However, after modification with SiO_2_-PEI@Ag nanocomposites, curve (b) showed a well-defined and significantly enhanced redox peak pair, confirming that the surface-bound Ag NPs provided the essential electroactive electron couples. Compared with pristine Ag NPs alone (Figure 5), the SiO_2_-PEI@Ag nanocomposite displayed a substantially higher peak current, sharper redox peaks, and a noticeable negative shift in the oxidation potential. Mechanistically, the ultra-small Ag NPs lower the electrode potential and accelerate dissolution kinetics via the nano-size effect, while the high-surface-area SiO_2_ core provides a large specific surface area for massive Ag NPs loading, effectively prevents Ag NPs aggregation during the oxidative dissolution process, and endows the interface with a highly efficient charge transfer capability [35,45,46]. Subsequently, compared with curve (b), the sequential immobilization of the anti-MPA antibody (curve (c)) and the BSA blocking (curve (d)) both led to a progressive and marked decrease in the peak currents, as these introduced biomacromolecules acted as non-conductive barriers that hindered interfacial electron transfer. Finally, when MPA was introduced, curve (e) displayed a further decrease in current response because the specific formation of antibody–antigen immunocomplexes created enhanced steric hindrance and continuously impeded electron transfer at the interface, successfully verifying the specific recognition capability of the fabricated biosensor.

The observed current variation is directly related to the change in the charge-transfer resistance (Rct) at the electrode interface, which is intrinsically influenced by the surface coverage of the electrode [31]. EIS was employed to evaluate the stepwise assembly of the electrochemical immunosensor. EIS measurements are typically presented as Nyquist plots, where the data forms a semi-circular trace characteristic of a charge-transfer limited process. The diameter of the semi-circular trace directly reflects the magnitude of the Rct [24,47]. As demonstrated in Figure 4B, compared with the bare gold electrode (curve (a)), the Rct of the SiO_2_-PEI@Ag NPs modified electrode (curve (b)) significantly decreased due to the excellent conductivity of Ag NPs. However, the Rct gradually increased as the electrode surface was sequentially modified with anti-MPA mAb, BSA, and MPA (curves (c–e)). The integrated results from both CV and EIS collectively verified the successful assembly of the proposed immunosensor.

Furthermore, the electrochemical characterization of the immunosensor was further evaluated by CV at different scan rates (Figure 4C). As the scan rate increased from 25 to 250 mV·s^−1^, the peak currents increased progressively, as shown in Figure 4(C1). Moreover, Figure 4(C2) shows that both anodic and cathodic peak currents depend linearly on the square root of the scan rate, with correlation coefficients (R^2^) of 0.9894 and 0.9836, respectively. These results indicated that the redox reaction was governed by the solid-state electrochemical dissolution of the surface-bound Ag NPs, accompanied by the dynamic interfacial ion mass transfer during the continuous potential scans.

### 3.3. Optimization of Experimental Conditions for Electrochemical Detection

**Figure 6:**
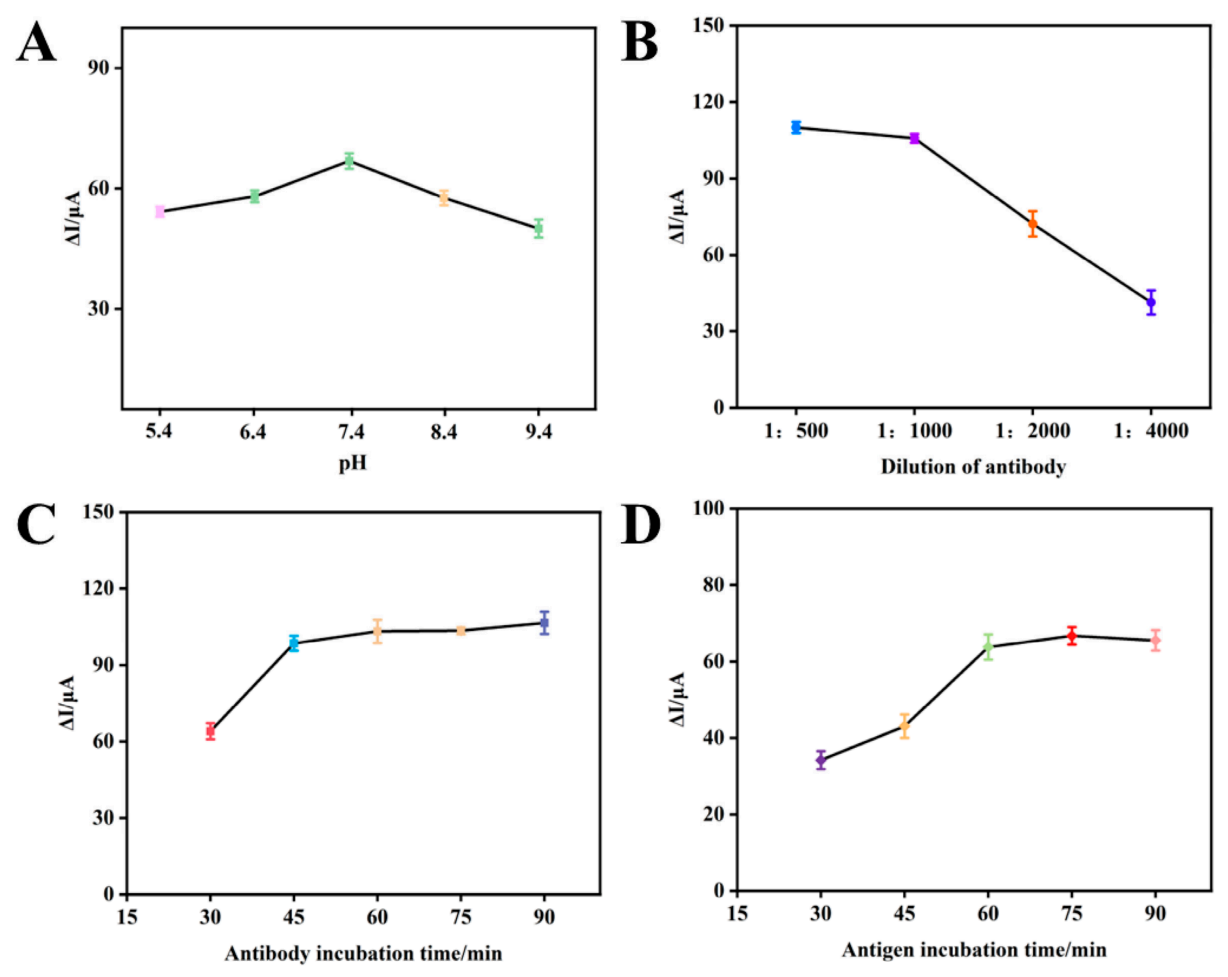
Optimization of experimental conditions for electrochemical detection. The pH of PBS (**A**), the dilution of anti-MPA mAb (**B**), the incubation time of the antibody (**C**), and the incubation time of the antigen (**D**). Note: ΔI = I_0_ − I_i_, where I_0_ and I_i_ are the peak currents measured in 0.1 M PBS before and after the specific capture of MPA, respectively.

Optimization of major experimental parameters is necessary to achieve the optimal performance of the electrochemical immunosensor. All optimization experiments were performed in 0.1 M PBS using DPV analysis, and the analytical performance was evaluated by monitoring the current difference (ΔI). The pH of the 0.1 M PBS plays a critical role in antigen–antibody binding and thus directly impacts the sensitivity of the immunosensor. As shown in Figure 6A, the current difference (ΔI) of the immunosensor initially increased and then decreased as the PBS pH varied from 5.4 to 9.4, reaching its maximum at pH 7.4. Consequently, all subsequent experiments were carried out at pH 7.4 to achieve optimal analytical performance, as this condition preserves antibody bioactivity and ensures a stable electrochemical response.

As shown in Figure 6B, the dilution of the anti-MPA antibody had a notable influence on the immunosensor response. DPV measurements indicated that antibody dilutions of 1:500 and 1:1000 generated comparable current difference (ΔI), whereas further dilution to 1:2000 or 1:4000 resulted in a marked decrease in signal intensity. Therefore, the antibody dilution of 1:1000 was adopted for subsequent experiments, as it ensured high sensitivity while reducing antibody consumption.

Optimization of the incubation time for both the anti-MPA antibody and the MPA antigen was crucial for enhancing the immunosensor performance. Figure 6C shows that the current difference (ΔI) increased with antibody incubation time and reached a plateau at 45 min, indicating saturation of antibody binding on the electrode surface. Similarly, the effect of antigen incubation time was investigated. As shown in Figure 6D, the current difference (ΔI) increased rapidly at the initial stage and then leveled off at 60 min, indicating that 60 min was sufficient for effective antigen–antibody binding. Therefore, optimal incubation times of 45 min and 60 min were established for the anti-MPA antibody and MPA antigen, respectively.

### 3.4. Electrochemical Performance of the Immunosensor

**Figure 7:**
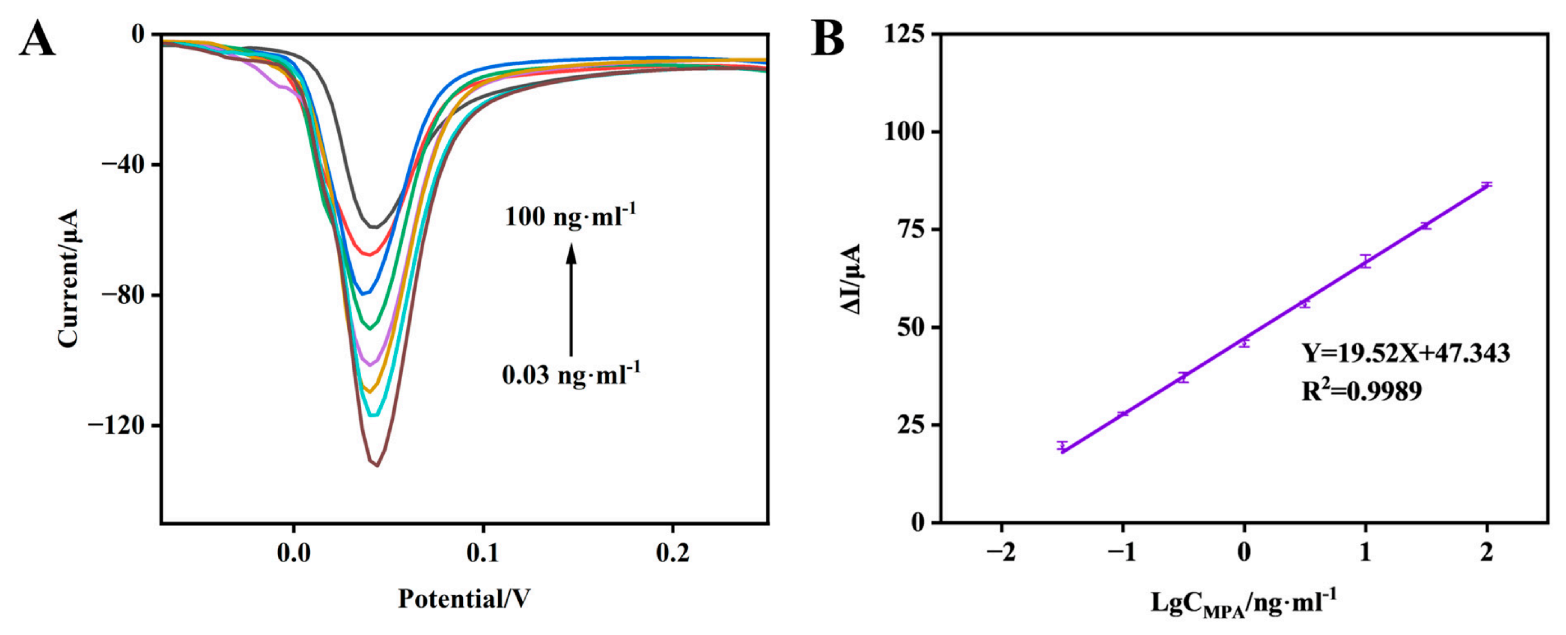
(**A**) DPV curves of the electrochemical immunosensor recorded at various MPA concentrations (0.03, 0.10, 0.32, 1, 3.16, 10, 31.62, and 100 ng·mL^−1^), where different colored lines represent different concentrations and the arrow indicates the trend of peak currents. (**B**) Calibration plots of ΔI versus log of MPA solution concentration (error bars = SD, *n* = 3). Note: ΔI = I_0_ − I_i_, where I_0_ and I_i_ are the peak currents measured in 0.1 M PBS before and after the specific capture of MPA, respectively.

Under the optimized conditions, the analytical performance of the constructed immunosensor was evaluated by DPV using MPA solutions at different concentrations. As shown in Figure 7A, the peak current decreased with increasing MPA concentration. Figure 7B shows a clear linear relationship between ΔI and MPA concentration in the range of 0.03–100 ng/mL, and the corresponding linear regression equation was determined to be ΔI = 19.52 LgC_MPA_ + 47.343 (where C_MPA_ represented the concentrations of MPA), with an R^2^ value of 0.9989. Statistical analysis revealed that the 95% confidence intervals (CI) for the slope and intercept were [18.872, 20.167] and [46.584, 48.102], respectively, confirming the high reliability of the linear regression. The limit of detection (LOD) was estimated to be 0.014 ng/mL based on 3 S_B_/m, where S_B_ means standard deviation of the blank and m means slope [27,29]. The proposed electrochemical immunosensor exhibited a wide linear range, low LOD, and high sensitivity among existing methods for MPA determination, as summarized in Table 1. Although a direct, standardized comparison of sensitivity among different methods is challenging due to the non-uniform units of LODs (e.g., ng/g versus ng/mL), distinct procedural variations in sample preparation are evident. Compared with chromatographic methods and certain traditional immunoassays that still rely on extensive cleanup, the analytical workflow proposed in this work significantly reduces the dependency on rigorous sample purification. Benefiting from the high specificity of the anti-MPA mAb and the high sensitivity of the electrochemical sensing platform, this method circumvents the need for tedious solid-phase extraction (SPE) and time-consuming solvent evaporation, requiring only basic protein precipitation and defatting procedures. Furthermore, owing to the intrinsic signal-amplification mechanism and the label-free feature that eliminates secondary antibody requirements, the constructed electrochemical immunosensor reduces both operational cost and analytical time compared with traditional immunoassays. Consequently, this platform can serve as a cost-effective and practical complement to standard routine methods for rapid, high-throughput screening. It should be mentioned that, as reflected in Table 1, whereas standard chromatographic techniques provide a well-defined limit of quantification (LOQ), the present study evaluated the LOD as a primary indicator of analytical performance. Although LOD determination is sufficient for rapid trace screening, LOQ evaluation remains to be conducted in future optimization.

### 3.5. The Selectivity, Reproducibility, and Stability of the Immunosensor

**Figure 8:**
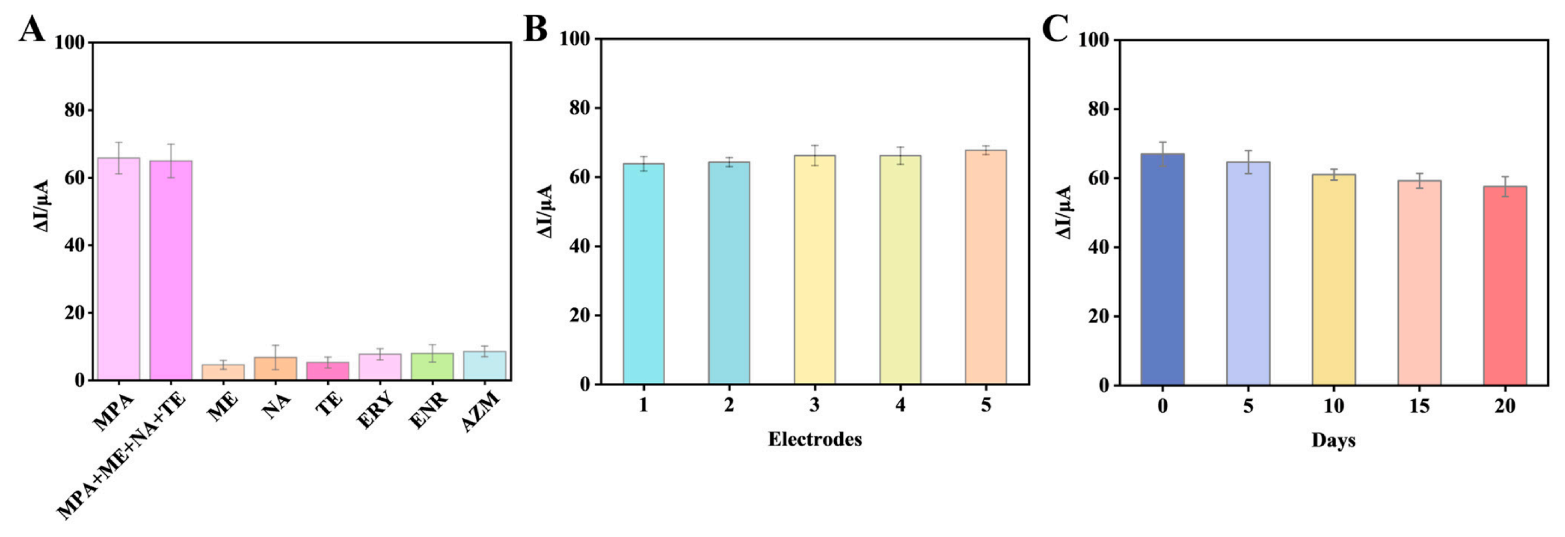
(**A**) Selectivity of the immunosensor toward medroxyprogesterone acetate (MPA, 10 ng/mL) against potential interferents, including 17-methyltestosterone (ME), flurogestone acetate (NA), testosterone (TE), erythromycin (ERY), enramycin (ENR), and azithromycin (AZM) (50 ng/mL each). (**B**) Reproducibility of the immunosensor based on five independently prepared electrodes under identical conditions. (**C**) Stability of the immunosensor evaluated by monitoring current responses at different storage times (error bars = SD, *n* = 3). Note: ΔI = I_0_ − I_i_, where I_0_ and I_i_ are the peak currents measured in 0.1 M PBS before and after the specific capture of MPA, respectively.

Selectivity, reproducibility and stability are key criteria for evaluating the performance of the proposed immunosensor. To assess selectivity, interference experiments were conducted using structurally related steroid hormones and common antibiotics as potential interfering compounds, including 17-methyltestosterone (ME), flurogestone acetate (NA), testosterone (TE), erythromycin (ERY), enramycin (ENR), and azithromycin (AZM), and the concentrations of these interferents were set at fivefold higher than that of MPA. As shown in Figure 8A, the tested interfering compounds induced negligible changes in peak current, whereas the mixture of structural analogues containing MPA (10 ng/mL) produced a current response comparable to that of MPA alone at the same concentration. These results indicate that the constructed immunosensor possesses high selectivity toward MPA, which can be attributed to the specific recognition between MPA and the immobilized anti-MPA antibody.

Reproducibility of the immunosensor was evaluated using five modified electrodes prepared under identical experimental conditions. As shown in Figure 8B, the relative standard deviation (RSD) among these sensors was 3.52%, and the RSDs within the groups were 3.27%, 2.03%, 4.37%, 3.80%, and 1.84%, respectively. In addition, the analytical precision was systematically evaluated by analyzing three concentrations of MPA standard solutions (100, 10, 1 ng/mL) in triplicate (*n* = 3). The results showed that the intra-day RSDs ranged from 2.65% to 4.51%, while the inter-day RSDs ranged from 5.59% to 7.50% (Table 2). These low RSD values confirm the high accuracy and reliable analytical precision of the sensor.

Furthermore, the immunosensors were stored in 0.1 M PBS buffer at 4 °C, and their stability was evaluated by DPV measurements at five-day intervals. As shown in Figure 8C, the current response exhibited only a 3.42% decrease after 5 days, and retained 91.17%, 88.50%, and 85.95% of the initial signal after 10, 15, and 20 days, respectively. These results indicate that the proposed immunosensor possesses acceptable stability over the tested storage period. Therefore, these results demonstrate that the developed immunosensor possesses excellent analytical reliability and stability, serving as a reliable analytical tool for further practical applications.

### 3.6. Practical Application of the Immunosensor

In this work, the practical performance of the proposed immunosensor was further evaluated through recovery experiments using MPA-spiked samples. Blank pork and milk samples were spiked with MPA at three concentration levels, and these samples were prepared according to the previously reported methods. Subsequently, 20 μL of each spiked sample was applied to the electrode surface for analysis. As summarized in Table 3, the recoveries of spiked MPA in pork samples ranged from 91.32% to 97.29%, with RSDs of 4.79–11.35%. Similarly, the recoveries of spiked MPA in milk samples ranged from 95.14% to 102.54%, with RSDs of 1.71–8.09%. The acceptable recoveries and RSDs obtained for the spiked samples demonstrate that the proposed immunosensor is suitable for the accurate determination of MPA in real samples.

However, it should be noted that naturally contaminated positive samples were not tested in this stage. Furthermore, a direct parallel comparison with the national standard method (GB 31660.4-2019) remains to be conducted in further verification studies. This reflects a common limitation in current electrochemical biosensor research. Although electrochemical immunoassays have garnered extensive attention and exhibit compelling potential for rapid screening, they currently remain unapproved by regulatory authorities for routine monitoring programs. This status is primarily attributed to the persisting lack of universal standardization and rigorous inter-laboratory validation protocols across the field. Overcoming these procedural and manufacturing bottlenecks to transition these laboratory prototypes into standardized, officially recognized regulatory tools represents a critical challenge that requires collective efforts from the entire research community [26].

## 4. Conclusions

In this work, a highly sensitive electrochemical immunosensor based on SiO_2_-PEI@Ag NPs nanocomposites and an anti-MPA antibody was successfully developed for the detection of MPA residues in animal-derived foods. The enhanced analytical performance is primarily governed by the unique properties and synergistic effects of the components within the SiO_2_-PEI@Ag NPs nanocomposite. Specifically, the core–shell structure leverages the large specific surface area of SiO_2_ NPs to support the high-density assembly of Ag NPs, which play a pivotal role in providing excellent electrical conductivity and electrochemical activity, thereby facilitating accelerated electron transfer and substantial signal amplification. Benefiting from these features and the high affinity of the anti-MPA mAb, the constructed platform achieved a low LOD of 0.014 ng/mL and a wide linear detection range of 0.03–100 ng/mL for MPA. In addition, negligible cross-reactivity was observed toward structurally related steroid hormones and common antibiotics, including 17-methyltestosterone (ME), flurogestone acetate (NA), testosterone (TE), erythromycin (ERY), enramycin (ENR), and azithromycin (AZM). Furthermore, the satisfactory recoveries obtained from spiked sample analyses validate the practical feasibility of the method. Overall, the proposed electrochemical immunosensor offers a reliable and cost-effective analytical alternative, demonstrating clear potential as a practical tool for the rapid screening of MPA residues in animal-derived foods.

## Figures and Tables

**Table 1:** Comparison of various analytical methods for MPA determination.

Methods	LOD	LOQ	Liner Range	Samples	Sample Preparation	Ref.
LC-MS/MS	0.5 μg/kg	1 μg/kg	-	Animal-derived foods	Multi-step extraction and SPE cleanup	GB 31660.4-2019 [20]
UHPLC-QE HF HRMS	0.1 μg/kg	-	0.1–100 μg/kg	Milk	ACN/hexane extraction, SPE, and N_2_ evaporation	[48]
UPLC-MS/MS	0.2 ng/g	1.0 ng/g	-	Animal-derived matrix	LLE and EMR-lipid clean-up	[19]
ELISA	0.096 ng/g	-	0.1–8.1 ng/g	pork	LLE, and SPE	[49]
LFA	10 μg/kg	-	-	swine liver	LLE, vacuum evaporation, and SPE	[15]
AAS	0.25 ng/mL	-	0.63–39.8 ng/mL	Pork	Enzymatic hydrolysis, LLE, rotary evaporation, and SPE	[50]
Electrochemical immunosensor	0.01 ng/mL	-	0.03–100 ng/mL	Pork and milk	Protein precipitation, pH adjustment, and defatting	This work

Note: UHPLC-QE HF HRMS, ultra-high-performance liquid chromatography coupled with quadrupole-high field Orbitrap high-resolution mass spectrometry. LFA, lateral flow assays. AAS, atomic absorption spectrometry. LLE, Liquid–liquid extraction. SPE, solid-phase extraction.

**Table 2:** Intra-day and inter-day precision of the proposed immunosensor (*n* = 3).

Concentrations (ng/mL)	Intra-Day (ΔI, μA)				Inter-Day (ΔI, μA)			
	1	2	3	RSD (%)	1	2	3	RSD (%)
100	86.02	89.07	84.57	2.65	95.37	85.4	92.16	5.59
10	67.78	68.24	64.5	3.05	65.87	61.07	68.93	6.07
1	46.53	47.37	43.45	4.51	45.97	49.5	42.6	7.50

Note: ΔI = I_0_ − I_i_, where I_0_ and I_i_ are the peak currents measured in 0.1 M PBS before and after the specific capture of MPA, respectively.

**Table 3:** Immunosensor analysis of MPA in pork and milk samples.

Sample	Added (ng/mL)	Found (ng/mL)	Recovery (%)	RSD (%)
Pork	100	94.08	94.08	11.35
	10	9.73	97.29	9.80
	1	0.91	91.32	4.79
Milk	100	102.54	102.54	8.09
	10	9.87	98.75	3.07
	1	0.95	95.14	1.71

## Data Availability

The original contributions presented in this study are included in the article. Further inquiries can be directed to the corresponding author.
